# Pre-treatment ^68^ Ga-PSMA-11 PET/CT Prognostic Value in Predicting Response to ^177^Lu-PSMA-I&T Therapy and Patient Survival

**DOI:** 10.1007/s11307-024-01900-6

**Published:** 2024-02-15

**Authors:** Roya Eisazadeh, Seyed Ali Mirshahvalad, Gregor Schwieghofer-Zwink, Lukas Hehenwarter, Gundula Rendl, Simon Gampenrieder, Richard Greil, Christian Pirich, Mohsen Beheshti

**Affiliations:** 1 Division of Molecular Imaging and Theranostics, Department of Nuclear Medicine, University Hospital, Paracelsus Medical University Salzburg, Muellner Hauptstrasse 48, 5020 Salzburg, Austria; 2 Joint Department of Medical Imaging, University Medical Imaging Toronto (UMIT), University Health Network, Mount Sinai Hospital & Women’s College Hospital; University of Toronto, Toronto, ON Canada; 3 Department of Internal Medicine III With Haematology, Medical Oncology, and Oncologic Center, University Hospital, Paracelsus Medical University Salzburg, Salzburg, Austria

**Keywords:** Prostate cancer, PSMA, Positron emission tomography, Radioligand therapy, Theranostics, Lutetium

## Abstract

**Purpose:**

To assess the prognostic value of pre-treatment [^68^Ga]Ga-PSMA-11 PET/CT and other baseline clinical characteristics in predicting prostate cancer (PCa) patients response to [^177^Lu]Lu-PSMA (PSMA-I&T), as well as patient survival.

**Procedures:**

In this retrospective study, 81 patients who received [^177^Lu]Lu-PSMA-I&T between October 2018 and January 2023 were reviewed. Eligible patients had metastatic castration-resistant PCa, underwent pre-treatment [^68^Ga]Ga-PSMA-11 PET/CT, and had serum prostate-specific antigen (PSA) levels available. On PET/CT images, SUVmax, SULmax, SUVpeak, and SULpeak of the most-avid tumoral lesion, as well as SUVmean of the parotid gland (P-SUVmean) and liver (L-SUVmean), were measured. Also, whole-body PSMA tumour volume (PSMA-TV) and total lesion PSMA (TL-PSMA) were calculated. To interpret treatment response after [^177^Lu]Lu-PSMA-I&T, a composite of PSA values and [^68^Ga]Ga-PSMA-11 PET/CT findings were considered. The outcomes were dichotomised into progressive versus controlled (stable disease or partial response) disease. Then, the association of baseline parameters with patient response was evaluated. Also, survival analyses were performed to assess baseline parameters in predicting overall survival.

**Results:**

Sixty patients (age:73 ± 8, PSA:185 ± 371) were included. Patients received at least one cycle of [^177^Lu]Lu-PSMA therapy (median = 4). Overall, half of the patients showed disease progression. In the progressive versus controlled disease evaluation, the highest SULmax, as well as SUVmax and SULmax to both backgrounds (L-SUVmean and P-SUVmean), were significantly correlated with the outcome (*p*-values < 0.05). In the multivariate analysis, only SULmax to the L-SUVmean remained significant (*p*-value = 0.038). The best cut-off was 8 (AUC = 0.71). With a median follow-up of 360 days, 11 mortal events were documented. In the multivariate survival analysis, only SULmax to P-SUVmean (cut-off = 2.4; *p*-value = 0.043) retained significance (hazard ratio = 4.0).

**Conclusions:**

A greater level of PSMA uptake, specifically higher tumour-to-background uptake in the hottest lesion, may hold substantial prognostic significance, considering both [^177^Lu]Lu-PSMA-I&T response and patient survival. These ratios may have the potential to be used for PCa patient selection for radioligand therapy.

## Introduction

Prostate cancer (PCa) is the second most common malignancy in men worldwide, showing poor prognosis in patients with metastatic castration resistance prostate cancer (mCRPC) [1, 2]. However, [^177^Lu]Lu-PSMA therapy has shown to be an emerging treatment for mCRPC patients, with encouraging clinical outcomes [3–5]. The TheraP study, a randomized, open-label, phase 2 trial, demonstrated that patients receiving [^177^Lu]Lu-PSMA-617 had better serum prostate-specific antigen (PSA) responses and progression-free survival (PFS) compared to those receiving alternatives [6]. Moreover, the phase 3 VISION study showed that adding [^177^Lu]Lu-PSMA to standard care improved overall survival (OS) and imaging-based PFS in patients with mCRPC [7].

Thus, through these comprehensive investigations, it has been shown that [^177^Lu]Lu-PSMA is of benefit in mCRPC patients overall. However, it seems that since 20–30% of patients may not respond to this therapy, there is a need for predictors that can further prognosticate treatment response and optimize patient selection. This will maximize therapeutic efficacy, preventing the blind administration of this beneficial therapy to mCRPC patients in the clinic merely based on the diagnosis of mCRPC [8–11]. In this regard, several baseline factors, including laboratory and clinical parameters (*e.g.* serum Chromogranin A and lactate dehydrogenase levels, age, and pain experience), have been discussed to estimate the patient’s survival and response to treatment [12, 13]. Nevertheless, it is essential to note that these factors have some limitations in predicting response to treatment.

[^68^Ga]Ga-PSMA positron emission tomography/computed tomography (PET/CT) is a reproducible, robust modality playing a crucial role in the diagnosis and treatment planning of advanced PCa [14, 15]. Despite varying inclusion criteria based on baseline [^68^Ga]Ga-PSMA PET/CT across clinical trials and treatment facilities globally, experts recommended utilizing [^68^Ga]Ga-PSMA PET/CT for patient selection. Recent trials, such as VISION and TheraP, have employed different criteria to assess tumour uptake. In the VISION trial, qualitative thresholds were used to assess tumour uptake relative to liver uptake. The cut-off used in the TheraP trial for lesions’ maximum standardized uptake value (SUVmax) was 20, being significantly higher, approximately 2–3 times greater than liver uptake and relatively similar to the uptake observed in the parotid gland [6, 7]. Thus, although these trials have shown the efficacy of [^177^Lu]Lu-PSMA therapy in highly PSMA-avid patients based on their inclusion criteria, their findings may not be entirely compatible with our daily routine observations in the clinic to decide whether patients would *really* benefit from receiving [^177^Lu]Lu-PSMA or not.

Moreover, as another gap in the current literature, most of the previous studies only assessed treatment response by measuring biochemical changes, primarily PSA levels. While PSA reduction is widely used in clinics due to its simplicity, there is ongoing debate regarding its preciseness to be the best response evaluation criteria. Although the literature on imaging-derived predictors using pre-treatment [^68^Ga]Ga-PSMA-11 PET/CT is limited, existing studies demonstrated a correlation between high PSMA expression (as evaluated by SUVs) and favourable results [8, 15–17]. The utilization of molecular response assessment in PSMA-targeted imaging is currently under investigation and has recently been endorsed by a joint consensus [16]. A recent investigation showed that response evaluation criteria in PSMA PET/CT (RECIP) classification could be robust, not only quantitatively but also interpreted qualitatively [17]. Furthermore, Gafita et al. introduced PSA + RECIP as a novel composite-based approach for evaluating treatment response [18]. This composite response classification system (PSA + RECIP) had a higher prognostic accuracy for OS, being superior to relying solely on PSA measurements or RECIP criteria.

Hence, in this study, we aimed to assess the potential of pre-treatment [^68^Ga]Ga-PSMA-11 PET/CT, as well as other baseline characteristics, in predicting response to [^177^Lu]Lu-PSMA (PSMA-I&T), considering a composite of both PSA and pre-treatment [^68^Ga]Ga-PSMA-11 PET/CT based on the state-of-the-art response assessment framework [18]. To our knowledge, our study is the first to assess the prognostic value of pre-treatment PSMA PET/CT and baseline clinical characteristics to predict treatment response classified by this novel method. In addition, we performed survival analysis and evaluated the prognostic value of the baseline measurements to predict patients’ OS.

## Materials and Methods

### Study Population

**Fig. 1:**
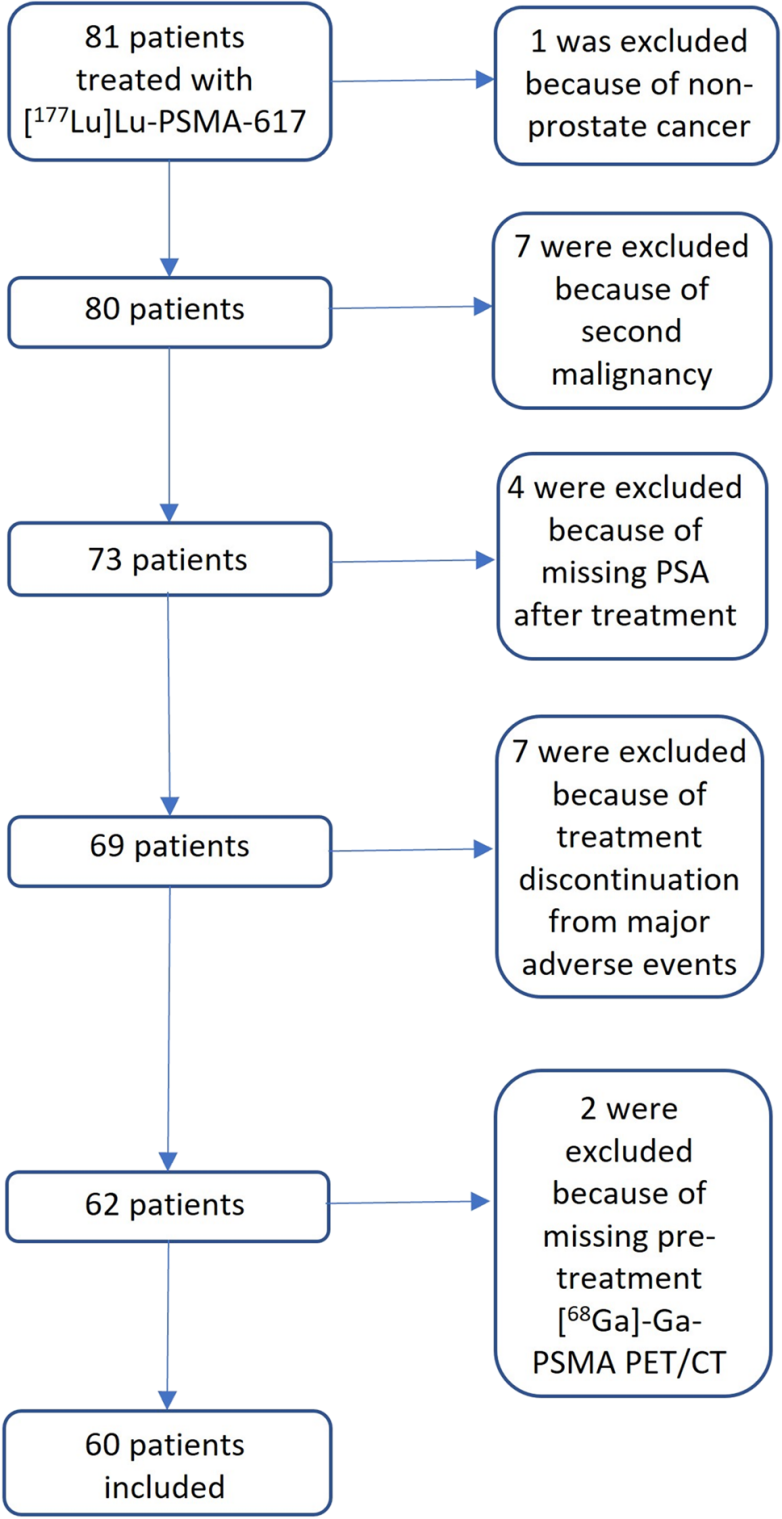
Patient selection flowchart

In this retrospective single-centre study, we identified 81 patients who received treatment with [^177^Lu]Lu-PSMA-I&T between October 2018 and January 2023. Eligible patients had mCRPC, underwent [^68^Ga]Ga-PSMA-11 PET/CT before treatment, and had serum PSA levels available at baseline and after each cycle of treatment. We excluded patients who discontinued treatment due to major adverse events such as renal failure or had a second malignancy. Finally, 60 patients were included (Fig. 1). Prior to PSMA radioligand therapy (RLT), all patients underwent standard second-line androgen deprivation therapy (ADT) with enzalutamide or abiraterone and third-line chemotherapy. Local radiotherapy and systemic [^223^Ra]Ra-dichloride RLT have been performed in 41 (68%) and 2 (3%) patients, respectively. Blood testing was carried out upon patient admission for every RLT session.

### PSMA Preparation and PET/CT Acquisition

The [^68^Ga]Ga-PSMA-11 was prepared using a commercially available cold kit (Telix Pharmaceuticals, Inc. Australia) and a commercial ^68^Ge/^68^Ga generator (Galli Ad®) manufactured in compliance with good manufacturing practices (GMP). Quality check was performed by thin-layer chromatography (TLC) to ensure radiochemical purity > 95%. The imaging was conducted systematically following standard procedure guidelines [19], which included scanning from the base of the skull to the proximal femur using two PET/CT scanners (Philips Ingenuity TF, Amsterdam/the Netherlands, and Siemens Biograph mCT, Erlangen/Germany). The [^68^Ga]Ga-PSMA-11 PET acquisition time was 2.5 min. per bed-position with a mean interval of 60 min (SD ± 14.4 min) between tracer administration and the start of the imaging. The mean injected activity per kg/body weight was 2.15 MBq. For attenuation correction and localization, a non-contrast-enhanced low-dose CT scan was performed (Siemens: Care Dose 4D, Care kV, slice thickness 1.2 mm and pitch 1.5; Philips: 100 kV, 33 mAs, slice thickness 1.5 mm and pitch 0.8). The reconstructed slice thickness was 3 mm, using iDose mode level 3 (Philips Ingenuity TF), respectively, SAFIRE level 3 (Siemens Biograph). Both PET/CT scanners are EARL/EANM accredited, and thus their performance is assumed to be similar.

### PET/CT Analysis and Interpretation

Quantitative analysis was performed using Syngo.via platform (Siemens Healthineers, Erlangen, Germany). The visual evaluation was conducted by two experienced nuclear medicine physicians, who reached diagnostic decisions through consensus. The readers were blind to the clinical information of patients, including serum PSA levels, and were only aware of PCa diagnosis. Structures with physiologic PSMA uptake (*e.g.* salivary glands) or known PSMA false-positive findings (*e.g.* celiac ganglia) were excluded. Lesions with visually higher uptake than the lumbar vertebral body were rated as PSMA-positive, indicating metastases [20]. Measurements of SUVmax, SULmax, SUVpeak, and SULpeak of the most-avid lesion, as well as SUVmean values for the parotid gland (P-SUVmean) and healthy liver tissue (L-SUVmean) as backgrounds, were taken using a standard volume region of interest (VOI).

Volumes of interests (VOIs) were delineated using isocontours set at two different thresholds: 45% of the maximum uptake [20] and a fixed SUVmax of 3 [21, 22]. These contours were drawn for all PSMA-positive lesions, and the contoured volumes were summed up for each patient. Subsequently, the PSMA tumour volume (PSMA-TV) and total lesion PSMA (TL-PSMA; calculated as PSMA-TV multiplied by SUVmean) were determined and reported at the aforementioned thresholds separately (generating PSMA-TV-45% and PSMA-TV-3, as well as TL-PSMA-45% and TL-PSMA-3). The number of metastatic lesions and prominent sites of the disease (prostate, lymph nodes, bone, and viscera) was also recorded.

### [^177^Lu]Lu-PSMA Therapy

[^177^Lu]Lu-PSMA-I&T was administered based on the recommendations of the multidisciplinary team, including board-certified nuclear medicine physicians, urologists, oncologists, radiologists, and pathologists. All patients received at least one cycle of [^177^Lu]Lu-PSMA-I&T RLT, with a mean interval of 6–8 weeks between consecutive cycles. The standard therapy protocol included 4–6 cycles of [^177^Lu]Lu-PSMA-I&T RLT unless patients revealed a major adverse event or showed a significant progression which resulted in therapy termination based on the multidisciplinary team consensus.

In each cycle, 7.4 GBq of [^177^Lu]Lu-PSMA I&T was administered, with a reduction of approximately 20% in activity if an individual exhibited decreased renal or haematological function. Prior to treatment infusion, each patient received intravenous hydration (500 mL 0.9% NaCl) and cooling of the salivary glands, starting 30 min before treatment infusion. The [^177^Lu]Lu-PSMA-I&T solution was administered intravenously by a perfusion system within 20 min.

### Response Assessment

All patients had serum PSA measurements after each cycle within 6–8 weeks. In the interpretation of treatment response, we interpreted the PSA values based on the prostate cancer working group 3 (PCWG3). Blindly to these interpretations and patients’ serum PSA levels, we evaluated [^68^Ga]Ga-PSMA-11 PET/CT findings based on RECIP (version 1.0). Finally, we synthesized these findings based on a novel framework for response evaluation criteria (PSA + RECIP) for those patients who underwent follow-up PET/CT imaging [18]. The final interpretation of patients’ response to therapy was made based on clear-cut definitions for each response group (partial response, PSA decline ≥ 50% or RECIP-PR; progressive disease, PSA increase ≥ 25% or RECIP-PD; stable disease, being stable in both evaluations). Detailed definitions are provided in Table 1. Then, we dichotomised the results into progressive versus controlled disease (stable disease or partial response), according to the patient’s outcome [16, 17].

**Table 1:** Response to treatment framework

Criteria	Definition
PCWG3 - PSA-PR - PSA-PD - PSA-SD	Decreasing PSA by more than 50% Increasing PSA by more than 25% Decreasing PSA by less than 50% or increasing PSA by less than 25%
RECIP1.0 - RECIP-PR - RECIP-PD - RECIP-SD	Decline ≥ 30% in PSMA-avid tumour volume and no appearance of new lesions Increase ≥ 20% in PSMA-avid tumour volume and appearance of new lesions Any condition but RECIP-PR or RECIP-PD
Composite response classifications - Partial response - Progressive disease - Stable disease	**PSA decline ≥ 50% or RECIP-PR** **PSA increase ≥ 25% or RECIP-PD** **Being stable in both evaluations**

*PCWG3* the prostate cancer working group 3, *PSA* prostate specific antigen, *PR* partial response, *PD* progressive disease, *SD* stable disease, *RECIP* response evaluation criteria in PSMA PET/CT, *CR* complete response

### Patient Follow-up

The follow-up period was determined starting from the date of the first RLT cycle. Typically, patients underwent monthly laboratory testing during this period. In cases where patients passed away during their treatment, the date of their death was recorded.

### Statistical Analysis

All parameters were analyzed at the patient level. Continuous and categorical variables were presented as mean ± standard deviation (SD) and frequency (%), respectively. The differences in the clinical and PET/CT parameters between response groups were evaluated using the chi-square test or Student’s *t*-test for the categorical or continuous variables, respectively. Next, we evaluated the association of the serum PSA and pre-treatment [^68^Ga]Ga-PSMA-11 PET/CT semi-quantitative parameters with the response to treatment using logistic regression. We tried to find a cut-off for SULmax based on the Youden index (maximization of the summation of sensitivity and specificity using receiver operating characteristic curves). The dichotomized variables entered the multivariate analysis to find the most significant predictor.

In the prognostic evaluation, the continuous variables that were significantly associated with the response were converted to categorical variables. Again, the Youden index was used for this conversion. Regarding OS, univariate analysis was performed using the Kaplan–Meier method. The significance of the difference was investigated using the univariate Mantel-Cox log-rank test. Significant parameters in the univariate analysis entered the multiple Cox regression and were provided with their hazard ratio. All data were gathered and analyzed using SPSS software (IBM, ver. 22). The statistical significance level was set at a two-sided *p*-value less than 0.05.

## Results

In this retrospective study, 60 male patients (mean age, 73 ± 8; average baseline pre-treatment PSA, 185 ± 371) were included. Patients received at least one cycle of [^177^Lu]Lu-PSMA therapy (mean cumulative dose of 338.4 ± 143.1 MBq/kg, ranging 59 − 702 MBq/kg), with a median of four cycles. The average interval between two consecutive cycles was 54 (± 9) days. Regarding the [^68^Ga]Ga-PSMA-11 PET/CT semi-quantitative parameters, the average (± SD) of the highest SUVmax, SULmax, SUVpeak, and SULpeak in the study population were 59.17 (± 47.67), 44.83 (± 37.3), 37.09 (± 31.29), and 28.38 (± 24.36), respectively. Additionally, the average (± SD) L-SUVmean and P-SUVmean were 4.54 (± 1.42) and 12.70 (± 4.11), respectively. Details are provided in Table 2.

**Table 2:** Comparison of the clinical data and pre-treatment semi-quantitative measurements between different response groups (*n* = 60)

	All patients	Progressive vs. non-progressive		
		Non-progressive (*n* = 30)	Progressive (*n* = 30)	*p*-value
Age	73 ± 8	74 ± 8	72 ± 8	0.672
ISUP grade group > 2	19 (63%)	19 (63%)	23 (77%)	0.260
ISUP grade group > 3	14 (47%)	14 (47%)	22 (73%)	0.035*
Therapy cycles > 2	27 (90%)	27 (90%)	19 (63%)	0.015*
Pre-treatment PSA	185.3 ± 371.0	112.9 ± 298.0	257.7 ± 424.8	0.132
PSA after 1^st^ cycle	398.5 ± 1694.1	71.6 ± 264.5	725.4 ± 2355.4	0.141
Hottest metastatic site (bone)	16 (53%)	16 (53%)	20 (67%)	0.365
Highest SUVmax	59.17 ± 47.67	70.97 ± 43.65	47.37 ± 49.28	0.054°
Highest SUVpeak	37.09 ± 31.29	44.41 ± 26.74	29.76 ± 34.14	0.069°
Highest SULmax	44.83 ± 37.31	54.36 ± 34.27	35.31 ± 38.24	0.047*
Highest SULpeak	28.38 ± 24.36	34.23 ± 21.15	22.54 ± 26.25	0.063°
SUVmax/Liver SUVmean	14.75 ± 13.28	18.60 ± 13.92	10.90 ± 11.60	0.024*
SULmax/Liver SUVmean	11.26 ± 10.57	14.39 ± 11.27	8.13 ± 8.95	0.020*
SUVmax/Parotid SUVmean	4.89 ± 3.93	6.04 ± 3.98	3.74 ± 3.60	0.023*
SULmax/Parotid SUVmean	3.73 ± 3.13	4.65 ± 3.19	2.80 ± 2.81	0.020*
PTV-3	306 ± 496	298 ± 521	314 ± 477	0.902
TLP-3	2814 ± 4234	3112 ± 4608	2516 ± 3881	0.590
PTV-45%	89 ± 147	77 ± 125	102 ± 167	0.511
TLP-45%	1243 ± 1831	1358 ± 1998	1127 ± 1674	0.630

Data are provided as mean ± SD or frequency (%)

*Statistically significant (*p*-value < 0.05)

°0.05 < *p*-value < 0.1

### Response Prediction in the Final Assessment

Overall, 30/60 (50%), 4/60 (7%), and 26/60 (43%) of patients showed disease progression, stable disease, and treatment response in the final assessment, respectively. Regarding the differences between progressive and controlled disease groups at pre-treatment, ISUP GG > 3, therapy cycles > 2, pre-treatment highest SULmax, SUVmax to backgrounds (parotid and liver), and SULmax to backgrounds (parotid and liver) significantly differed between the two groups. Details are provided in Table 3.

**Table 3:** Comparison of the clinical data and pre-treatment semi-quantitative measurements between different response groups (*n* = 60)

	Progressive vs. non-progressive		
	Non-progressive (*n* = 30)	Progressive (*n* = 30)	*p*-value
Age	74 ± 8	72 ± 8	0.672
ISUP grade group > 2	19 (63%)	23 (77%)	0.260
ISUP grade group > 3	14 (47%)	22 (73%)	0.035*
Therapy cycles > 2	27 (90%)	19 (63%)	0.015*
Pre-treatment PSA	112.9 ± 298.0	257.7 ± 424.8	0.132
PSA after 1^st^ cycle	71.6 ± 264.5	725.4 ± 2355.4	0.141
Hottest metastatic site (bone)	16 (53%)	20 (67%)	0.365
Highest SUVmax	70.97 ± 43.65	47.37 ± 49.28	0.054°
Highest SUVpeak	44.41 ± 26.74	29.76 ± 34.14	0.069°
Highest SULmax	54.36 ± 34.27	35.31 ± 38.24	0.047*
Highest SULpeak	34.23 ± 21.15	22.54 ± 26.25	0.063°
SUVmax/Liver SUVmean	18.60 ± 13.92	10.90 ± 11.60	0.024*
SULmax/Liver SUVmean	14.39 ± 11.27	8.13 ± 8.95	0.020*
SUVmax/Parotid SUVmean	6.04 ± 3.98	3.74 ± 3.60	0.023*
SULmax/Parotid SUVmean	4.65 ± 3.19	2.80 ± 2.81	0.020*
PTV-3	298 ± 521	314 ± 477	0.902
TLP-3	3112 ± 4608	2516 ± 3881	0.590
PTV-45%	77 ± 125	102 ± 167	0.511
TLP-45%	1358 ± 1998	1127 ± 1674	0.630

Data are provided as mean ± SD or frequency (%)

*Statistically significant (*p*-value < 0.05)

°0.05 < *p*-value < 0.1

### Association between [^68^Ga]Ga-PSMA-11 PET/CT parameters and the response to treatment

In the progressive versus controlled disease evaluation, the highest SULmax (*p*-value = 0.046), highest SUVmax to the L-SUVmean (*p*-value = 0.024), highest SULmax to the L-SUVmean (*p*-value = 0.021), highest SUVmax to the P-SUVmean (*p*-value = 0.023), and highest SULmax to the P-SUVmean (*p*-value = 0.020) were significantly correlated with the outcome. In the multivariate analysis, only the highest SULmax to the L-SUVmean was significant (*p*-value = 0.038). The highest SULmax to the L-SUVmean had and AUC of 0.71 in determining controlled disease patients. The best cut-off was 8, showing a sensitivity of 67% and specificity of 74%.

### Response Prediction in the Fourth Cycle

In this step, we limited the response to treatment prediction to the responses achieved in patients with ≥ 4 cycles of [^177^Lu]Lu-PSMA therapy (*n* = 46) and, again, calculated the differences in the variables noted previously in Table 3. The details are provided in Table 4.

**Table 4:** Comparison of the clinical data and pre-treatment semi-quantitative measurements between different response groups at the 4^th^ cycle of ^177^Lu-PSMA therapy (*n* = 46)

	Progressive vs. non-progressive		
	Non-progressive (*n* = 30)	Progressive (*n* = 16)	*p*-value
Age	74 ± 8	72 ± 8	0.672
ISUP grade group > 2	19 (63%)	14 (88%)	0.083°
ISUP grade group > 3	14 (47%)	14 (88%)	0.007*
Pre-treatment PSA	127.8 ± 303.9	86.7 ± 151.4	0.615
PSA after 1^st^ cycle	86.4 ± 272.7	90.4 ± 129.8	0.956
Hottest metastatic site (bone)	16 (53%)	11 (69%)	0.373
Highest SUVmax	78.88 ± 58.50	39.26 ± 18.64	0.002*
Highest SUVpeak	49.90 ± 38.54	23.05 ± 9.91	0.001*
Highest SULmax	60.90 ± 45.83	27.99 ± 12.72	0.001*
Highest SULpeak	38.49 ± 29.97	16.99 ± 7.28	0.001*
SUVmax/Liver SUVmean	20.36 ± 16.15	9.25 ± 6.11	0.002*
SULmax/Liver SUVmean	15.86 ± 12.94	6.57 ± 4.08	0.001*
SUVmax/Parotid SUVmean	6.72 ± 4.75	2.77 ± 1.16	< 0.001*
SULmax/Parotid SUVmean	5.20 ± 3.78	1.98 ± 0.82	< 0.001*
PTV-3	339 ± 546	121 ± 159	0.049*
TLP-3	3547 ± 4902	963 ± 1235	0.010*
PTV-45%	82 ± 126	37 ± 38	0.078°
TLP-45%	1515 ± 2056	452 ± 536	0.011*

Data are provided as mean ± SD or frequency (%)

*Statistically significant (*p*-value < 0.05)

°0.05 < *p*-value < 0.1

In this population, the highest SUVmax (*p*-value = 0.013), highest SUVpeak (*p*-value = 0.010), highest SULmax (*p*-value = 0.008), highest SULpeak (*p*-value = 0.008), TL-PSMA-3 (*p*-value = 0.044), and TL-PSMA-45% (*p*-value = 0.048) were significant parameters in distinguishing progressive from controlled-disease patients. In the multivariate analysis, only SULmax to the P-SUVmean (*p*-value = 0.010) remained significant. In the search for the best cut-off for SULmax to the P-SUVmean to determine patients with controlled disease, 2.7 was the best, showing a sensitivity of 67% and a specificity of 88% (AUC = 0.83).

**Fig. 2:**
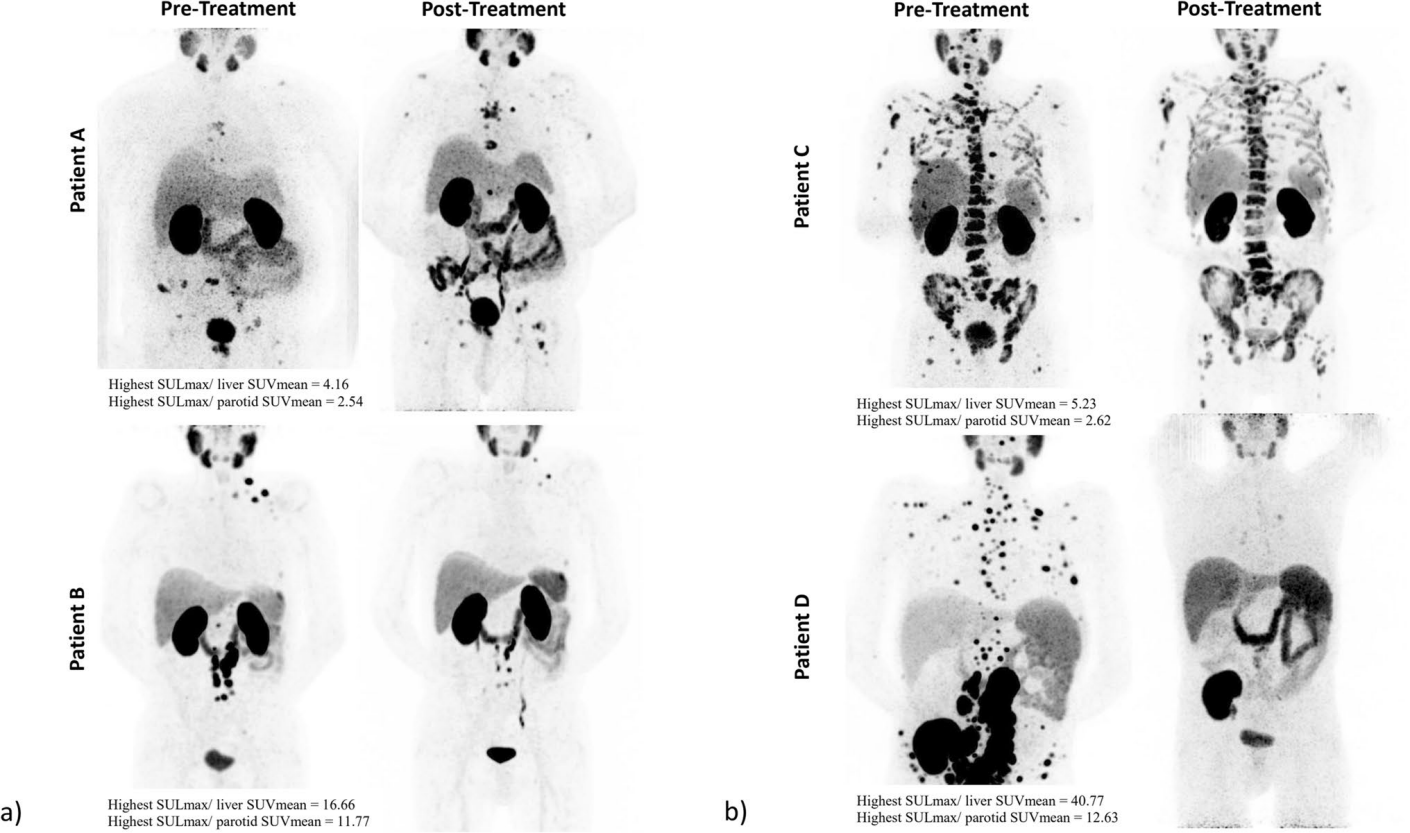
[^68^Ga]Ga-PSMA-11 PET/CT (MIP-maximum intensity projection images) of representative cases with **a** a low tumour burden (patients A and B) and **b** a high tumour burden (patients C and D). Patient A exhibits a low SULmax-to-parotid ratio (< 2.7), while patient B was categorized in the high group (≥ 2.7). Therefore, despite having a low tumour burden, patient A did not respond to the treatment. Patient D demonstrates a high SULmax-to-parotid ratio (≥ 2.7), while patient C was categorized in the low group (< 2.7). Consequently, despite having a high tumour burden, patient D responded to the treatment. Noteworthy, pre/post-therapy serum PSA values for these patients were 6.54/13.94, 4.83/0.45, 880.55/1728.21, and 576.49/46.11, respectively

To visualize the SULmax to backgrounds’ cut-off application in the response prediction, some cases are provided (Fig. 2). A higher SULmax ratio resulted in a better response to [^177^Lu]Lu-PSMA therapy.

### Survival Analysis (OS Prediction)

**Fig. 3:**
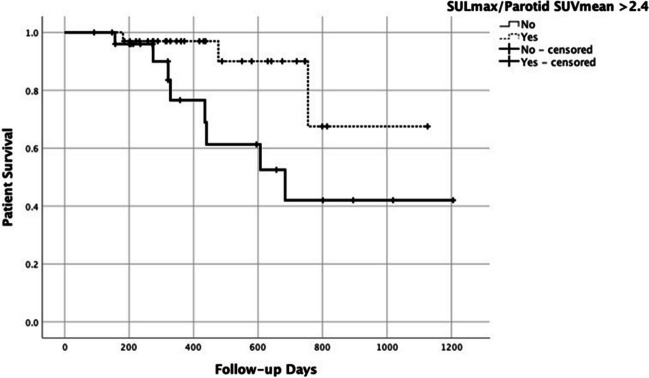
Kaplan–Meier survival plot for the highest SULmax to parotid background SUVmean (cut-off = 2.4; hazard ratio = 4)

With a median follow-up of 360 days (range: 91–1114 days), 11/60 (18%) mortal events were documented. Using the binarized variables, SUVpeak, SULmax, SUVmax to the L-SUVmean, SUVmax to the P-SUVmean, SULmax to the L-SUVmean, SULmax to the P-SUVmean, TL-PSMA-3, and TL-PSMA-45% had *p*-values < 0.1 (Table 5). In the multivariate analysis among the pre-treatment [^68^Ga]Ga-PSMA-11 PET/CT parameters, only the highest SULmax to P-SUVmean (cut-off = 2.4, *p*-value = 0.043) was significant (HR = 4.0; 95%CI = 1.1–15.0; Fig. 3).

**Table 5:** Detailed results of the survival analysis; [^68^ Ga]Ga-PSMA PET/CT parameters to predict overall survival

Variable	Cut-off	Hazard ratio (95%CI)	*p*-value
SUVpeak	25	3.8 (1.1–14.2)	0.036*
SULmax	30	3.5 (1.0–13.2)	0.049*
SUVmax-to-liver	10	3.1 (0.8–11.7)	0.082
SUVmax-to-parotid	3.3	3.6 (1.0–13.5)	0.045*
SULmax-to-liver	5.2	2.7 (0.8–9.4)	0.095
SULmax-to-parotid	2.4	4.0 (1.1–15.0)	0.029*
TLP-3	1400	3.0 (0.8–11.2)	0.092
TLP-45%	550	3.0 (0.8–11.2)	0.092

*Statistically significant (*p*-value < 0.05)

## Discussion

In this study, we evaluated the prognostic value of [^68^Ga]Ga-PSMA-11 PET/CT parameters alongside other clinical factors for predicting response to treatment, as well as OS, in mCRPC patients who received [^177^Lu]Lu-PSMA-I&T therapy. Treatment response, considered as combined molecular imaging and biochemical response (PSA + RECIP), was assessed in two categories: after the termination of all cycles (final assessment) and after completing the 4th cycle of treatment (the minimum recommended cycles to reach therapy efficacy). We found that the highest SULmax to the L-SUVmean had the highest AUC in detecting controlled disease, with a best cut-off value of 8. In the fourth cycle assessment, SULmax to the P-SUVmean was the only significant variable in the multivariate analysis to predict controlled disease, with a best cut-off value of 2.7. Notably, tumour volume or site of disease did not predict response to treatment. In the survival analysis, the highest SULmax to the P-SUVmean (cut-off = 2.4) was the only significant variable in the multivariate analysis to predict OS.

Regarding the clinical parameters predicting response to treatment, the study conducted by Ferdinandus et al. revealed that younger age and higher Gleason scores had a negative impact on treatment response [13]. However, our findings did not reach significance in relation to these parameters. On the other hand, they demonstrated that basal PSA did not reach significance as a predictor, which was in line with our results [13]. Similarly, Rathke et al. showed that baseline PSA had no significant prognostic value for predicting treatment response [12]. Considering the value of PSMA PET/CT-derived factors, our findings were consistent with the results of a study conducted by Emmett et al. [8] on PSMA PET/CT predictive parameters for response assessment in a limited cohort of 14 patients. Similarly, they revealed that maximal PSMA intensity is a reliable predictor of response to treatment. However, our study expanded on these findings by including a larger sample size and evaluating additional PET/CT parameters such as SUVmax, SULmax, and their ratio to background. In another study by van der Sar et al. they also demonstrated a significant correlation between the SUV of the most-avid metastases and response to treatment [23].

The other valuable finding in our study could be the less importance of the site of the metastases in therapy response. As we have seen in our routine clinic, sometimes there is a concern about the difference in the objective response among various metastatic patients (*e.g.* bone or visceral involvement). There are also reports that visceral metastasis can have a negative impact on patients’ disease course [9, 24]. However, our results, like some other previous studies [8, 23], demonstrated no significant difference between sites of metastasis, at least when compared to other prominent factors such as maximal PSMA uptake. Moreover, PET-based parameters like PSMA-TV and TL-PSMA, although significantly different between groups (in the fourth cycle assessment), did not keep their level of significance when compared to the more significant parameters (*e.g.* tumour-to-background ratios) in the therapy response prediction. A study conducted by Widjaja et al. was consistent with our findings, observing that PSMA expression from pre-therapeutic PET/CT exhibited superior performance compared to PSMA-TV and TL-PSMA [25]; though in the clinic, larger tumour volumes may initially seem to be associated with lower likelihoods of treatment response. Hence, distinguishing between metastatic patients based on their sites of metastases or solely based on pre-treatment involvement volumes may not be of a high value for patient selection prior to RLT, comparing them relative to the intensity of the involvement.

Considering OS, the percentage of registered mortal events in our study was more or less similar to the reported findings in the literature [26, 27]. Our results showed that the hottest lesion SULmax to P-SUVmean could predict patient survival. In the most recent international multi-centre study [28], similar to our findings, authors revealed that this ratio (notably, *SUVmean* to the P-SUVmean) was prognostic for therapy response and patient survival. They used the cut-off of > 1.5 to predict a higher survival rate in their study, being lower than our 2.7 coordinate point, which could be, to some extent, due to using lesions’ SULmax in our study instead of SUVmean. Notably, it has been shown that the tumour-to-background ratio could also be predictive of PFS [5]. In another recent study by Karimzadeh et al. they demonstrated that adhering to the patient selection criteria outlined in TheraP (PSMA-positive disease with a minimum SUVmax of 20 at the site of disease and SUVmax greater than 10 at all other sites of measurable metastatic disease) resulted in improved treatment responses and overall outcomes [29]. Notably, their inclusion criteria closely mirrored the criteria based on the parotid uptake threshold, which may suggest that our findings can be in alignment with the conclusions of their study in this regard. Although we followed a different methodology than TheraP, we also showed that highly intense uptake, more than backgrounds, can help identify responsive patients, and intense uptake, higher than parotid tissue, can predict better OS when patients are treated by RLT. Thus, their higher response rate than us can be justified by our different selection criteria. Moreover, previous studies noted that PSA level (even PSA doubling time), Gleason score, and sites of metastases cannot predict patient survival [30, 31]. This can be of importance when selecting patients to benefit from [^177^Lu]Lu-PSMA-I&T therapy, not excluding patients with high levels of PSA with a presumption of their highly extensive/aggressive disease.

This study suffered from some limitations, the most important being the inclusion of a rather small heterogeneous patient cohort receiving various cycles of [^177^Lu]Lu-PSMA-I&T therapy. To address this problem to some extent, we reselected patients who underwent at least four cycles of therapy to re-evaluate the studied parameters. However, this was a double-edged sword; although it reduced the heterogeneity to some extent, it made the study population smaller at the same time. The other limitation would be the retrospective design of the study, which may affect the findings, particularly the assessment of some laboratory parameters (*e.g.* alkaline phosphatase, Chromogranin A). Moreover, the statistical analysis might be affected by multiple comparisons. Also, calculating the SULmax-to-SUVmean (background) ratio could be of some concern because of the different nature of SUL and SUV based on body surface area adjustment, though it was a more robust predictor than SUV-to-SUV ratios and could retain its significance alongside other variables in the multivariable analyses. So, we decided to report our own experience and recommend further investigations in this regard. Regarding OS, in the survival analysis, we could not follow all patients for too long so that we could register all mortal events, resulting in patient censoring because of follow-up termination. Lastly, we did not include the response to the therapy class of the patients in our survival analysis. Although this may be considered a limitation, we intentionally did that in order to report helpful factors prior to treatment initiation to guide patient selection.

In conclusion, our study showed that higher PSMA uptake, best represented as high tumour-to-background uptake in the hottest lesion, can be of the most significant prognostic value in PCa patients receiving [^177^Lu]Lu-PSMA-I&T therapy. Additionally, we showed that a higher tumour-to-background ratio is associated with improved patient survival, which might possibly result from their better response to the treatment. These ratios can also be used for more robust patient selection, including subjects who are more likely to benefit from novel combination therapies. Future long-term prospective studies are strongly recommended to enhance the reported cut-offs for patient selection.

## Data Availability

The detailed data generated during and/or analyzed during the current study are available from the corresponding author upon reasonable request.
